# A Systematic Review of In Vivo Studies of the Efficacy of Herbal Medicines for Anti-Aging in the Last Five Years

**DOI:** 10.3390/ph16030448

**Published:** 2023-03-16

**Authors:** Seung-Yeon Cho, Han-Gyul Lee, Seungwon Kwon, Seong-Uk Park, Woo-Sang Jung, Sang-Kwan Moon, Jung-Mi Park, Chang-Nam Ko

**Affiliations:** 1 Stroke and Neurological Disorders Center, Kyung Hee University College of Korean Medicine, Kyung Hee University Hospital at Gangdong, Seoul 05278, Republic of Korea; 2 Department of Cardiology and Neurology, Kyung Hee University College of Korean Medicine, Kyung Hee University Medical Center, Seoul 02447, Republic of Korea

**Keywords:** anti-aging, aging, senescence, herbal medicine, review, in vivo studies, mechanisms of action

## Abstract

Background: The world’s population is rapidly aging, and attention to and research on the increase in life expectancy and age-related diseases are needed. This study aimed to review the in vivo studies on the anti-aging effects of herbal medicines. Methods: In vivo studies of single or complex herbal medicines for anti-aging that were published in the last five years were included in this review. The following databases were used: PubMed, Scopus, ScienceDirect, Web of Science and EMBASE. Results: A total of 41 studies were considered eligible for the review. The articles were classified into body organs and functions, experimental country, herbal medicine, extraction method, administration route, dosage, duration, animal model, aging-induced method, sex, number of animals per group, and outcomes and mechanisms A single herbal extract was used in a total of 21 studies including *Alpinia oxyphylla Miq.*, *Acanthopanax senticosus* and *Lyceum barbarum*, and a multi-compound herbal prescription was used in a total of 20 studies, including Modified Qiongyu paste, Wuzi Yanzong recipe, etc. Each herbal medicine had anti-aging effects on learning and memory, cognition, emotion, internal organs, gastrointestinal tracts, sexual functions, musculoskeletal function and so on. The common mechanisms of action were antioxidant and anti-inflammatory, and various effects and mechanisms for each organ and function were identified. Conclusions: Herbal medicine exhibited beneficial effects on anti-aging in various parts of the body and its function. Further investigation of the appropriate herbal medicine prescriptions and their components is recommended.

## 1. Introduction

According to the World Health Organization (WHO), by 2050, 22% of the world’s population (approximately 2 billion people) will be over the age of 60, and the problem of deteriorating health in old age is expected to increase significantly, owing to the increase in life expectancy and age-related diseases. Increased life expectancy and the desire for “healthy aging” to maintain good health in old age for as long as possible have led to the recognition of aging as a treatable disease, and interest in the field of research called “Anti-aging Medicine” is growing [1].

The WHO recently recognized aging as a “major disease risk factor” rather than a “natural phenomenon” and changed the “Senility (R54)” code of the 10th edition of the International Classification of Diseases (ICD-10) to the “Old age (MG2A)” code. As the ICD code is a prerequisite for the registration of all new drugs and treatments, it can be considered as laying the groundwork for the development of therapeutic interventions and prevention strategies targeting aging and age-related diseases.

Herbal medicine is attracting attention as it can play a role as an anti-aging treatment, and various experimental studies have been conducted on herbal medicines, such as ginseng [2,3,4,5]. Several studies have been conducted to identify the anti-aging effects and mechanisms of herbal medicines, and several reviews have been reported [6,7]. However, these papers do not contain any recently published articles, or else there are limitations in the studies themselves, one such being that they analyze only a few herbal medicines and ingredients.

Therefore, this study aimed to review research papers pertaining to the in vivo use of herbal medicines against aging, which have been published within the last five years. Specifically, studies conducted on rodents were chosen in order to find a single herbal medicine or else multi–compound herbal prescription that could be used in the clinic. Previous in vivo studies for anti-aging were diverse in research methods. Here, we summarize the results of the experiments on the available experimental models, evaluation parameters and mechanisms of herbal medicine available for anti-aging to provide a basis for additional in vivo research.

## 2. Materials and Methods

This systematic review was performed according to the recommendations of the Preferred Reporting Items for Systematic Reviews and Meta-Analysis (PRISMA) statement. All steps were conducted independently by two reviewers. A third reviewer was consulted in case of discrepancies. This study was registered in the Open Science Framework (OSF) with the registration DOI https://doi.org/10.17605/OSF.IO/KVHQF (accessed on 7 March 2023).

### 2.1. Search Strategy

A search strategy was first performed on 1 December 2021 and updated on 20 January 2023 in the following databases: PubMed, Scopus, ScienceDirect, Web of Science and EMBASE. The search query used was as follows: (herbal medicine) AND (antiaging OR senescence) AND (rat OR mice). References were organized in EndNoteTM^20^, and duplicates were removed in the same program. Screening was performed based on information in the titles and abstracts.

### 2.2. Inclusion and Exclusion Criteria

All available in vivo studies that assessed the anti-aging effects of herbal medicines were included in our review. The inclusion criteria were articles that were published within 5 years, in English, used rodent models (mice or rats) and assessed the anti-aging effects of single herbal extracts or complex herbal medicines. The exclusion criteria were review articles, conference abstracts, clinical trials, specific disease-induced model studies (e.g., Alzheimer’s disease, UVB-induced photoaging, etc.), studies performed in silico or in vitro, studies performed with only some ingredients or compounds isolated from herbs and studies on foods not used for therapeutic purposes in the clinic.

### 2.3. Data Collection Process

Data were collected using customized data extraction in Microsoft Excel^TM^ with the following data: first author, year of publication, publication journal, country, herbal medicine (plant part), type of extraction, route of administration, dose per day, treatment period, type and age of animal model (mice or rat), induced aging, sex, number of animals per group, outcomes and mechanisms.

## 3. Results

### 3.1. Study Selection

**Figure 1:**
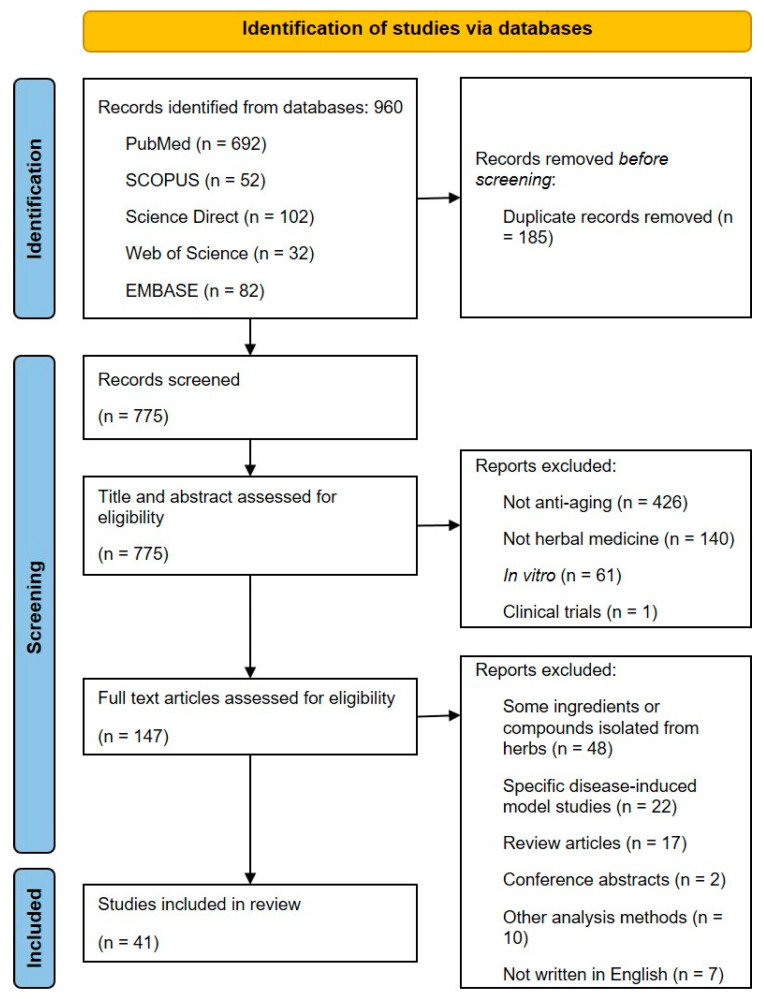
Flow diagram of the systematic review study selection process.

A total of 960 studies were screened in the initial electronic search, and 185 studies were excluded owing to duplicate publications. After screening the titles and abstracts, 628 studies were excluded for the following reasons: not anti-aging (n = 426), not herbal medicine (n = 140), in vitro (n = 61) and clinical trials (n = 1). After reviewing 147 full-text articles, 106 were excluded for the following reasons: studies performed with only some ingredients or compounds isolated from herbs (n = 48), specific disease-induced model studies (n = 22), review articles (n = 17), conference abstracts (n = 2), other analysis methods (e.g., network pharmacology, n = 10) and articles not written in English (n = 7). After this process, 41 studies were considered eligible for the review. A flow diagram of the article selection process is shown in Figure 1.

### 3.2. Characteristics of Included Studies

In total, 41 rodent studies using herbal medicines showed the efficacy or mechanisms of anti-aging. The classification, experimental country, herbal medicine, extraction method, administration route, dosage, duration, animal model, aging-induced method, sex, number of animals per group, and outcomes and mechanisms are summarized in Table 1.

The 41 studies were classified into the following categories: brain, learning and memory, cognition, emotion, cardiac function, liver and brain, liver, liver and kidney function, gastrointestinal tract, sexual function, musculoskeletal function, skin, skin and sexual organs, lifespan extension, physiology, gut microbiota and metabolome, and metabolomic analysis.

As for the countries where the experiments were conducted, China had the most cases (n = 20) [8,9,13,15,16,19,23,26,27,28,29,30,32,36,38,39,46,47,48,49], followed by Japan (n = 8) [12,20,40,41,42,43,44,45], Korea (n = 6) [24,31,33,34,35,37] and Taiwan (n = 3) [21,22,23]. Thailand [18], the United States [17], Mexico [11] and Poland [25] were each associated with one study.

There were 21 studies on single herbal extracts and 20 studies on multi-compound herbal prescriptions (Table 2). Among them, two studies used *Alpinia oxyphylla* Miq. as a single herbal medicine [21,22]. Three studies were conducted on the use of Guilingji [15,29,30], and two studies focused on Ninjin’yoeito (Ren-Shen-Yang-Rong-Tang in Chinese; Insamyangyung-tang in Korean) [41,45] as multi-compound herbal prescriptions.

In herbal medicine extraction, 17 studies used water extraction [9,13,14,17,20,21,22,27,31,33,39,40,42,45,47,48]; 6 studies used ethanol [10,16,18,24,34,35]; and 2 studies used carbon dioxide fluid [23,26]. Three studies used a suspension of the herbal formula Guilingji [15,29,30]. Methanol [12], water or volatile oil [19] and alcohol and water [28] were each used in one study. Ten studies did not report extraction methods.

The route of administration of herbal medicine was oral in 33 studies and intragastric in 7 studies [10,27,29,30,36,38,39]. One study did not report the route of administration [11]. Eighteen studies tested the dose of herbal medicine at a single concentration [11,13,17,18,19,20,21,24,28,31,37,38,39,40,42,46,48,50], and in 21 studies, 2 or 3 types of concentrations were tested [8,9,10,14,15,16,22,23,25,26,27,29,30,32,33,34,35,36,41,45,47]. The other two studies did not report the dosage used [12,44]. The duration of herbal medicine administration varied from 2 weeks to 2 years, but most ranged from 4 to 8 weeks.

The animals used in the experiments were Sprague-Dawley rats (n = 15) [11,13,14,15,16,18,22,24,29,30,32,33,34,35,47], C57BL/6 mice (n = 8) [8,10,28,31,36,44,45,46], Kunming mice (n = 4) [19,23,26,27], ICR mice (n = 3) [9,39,48], senescence-accelerated mouse-prone 8 (SAMP8) mice (n = 3) [12,20,40], BALB/c mice (n = 2) [37,38], hairless mice (n = 2) [42,43], CB6F1 mice (n = 1) [17], Swiss mice (n = 1) [25], Wistar Kyoto rats (n = 1) [21], Wild-type and NPY knockout mice (n = 1) [44] and Klotho-hypomorphic (kl/kl/) mice (n = 1) [41]. Regarding sex, five studies used both male and female mice or rats [17,27,29,43,44]; four studies used only female mice or rats [28,36,37,38]; and three studies did not mention the sex of the animals used [34,42,46]. The remaining studies used all male mice or rats.

Regarding the method of inducing aging, induction by d-galactose (D-gal) was performed in 14 studies [8,9,10,13,14,16,19,21,22,23,26,27,29,47], and natural aging was performed in 7 studies [28,30,36,38,42,43,48]. D-gal and NaNO_2_ [39], and 3-nitropropionic acid or doxorubicin injection [44] were each used in one study. The remaining studies did not mention the method of inducing aging.

Several of the studies identified antioxidant enzymes, such as superoxide dismutase (SOD), catalase (CAT) and glutathione peroxidase (GPX), as well as cytokines, such as interleukin-6 (IL-6) and tumor necrosis factor-α (TNF-α). In addition, the evaluation indicators and mechanisms were specific to each organ or function. The anti-aging effect on the brain (learning and memory, and cognition) was confirmed, mainly through memory function tests and observation of hippocampal neurogenesis. In studies on internal organs, the functions of each organ, blood tests and cytokines were observed. Studies related to the gastrointestinal tract were characterized by observing changes in the gastric histomorphology or intestinal flora. Most studies have confirmed changes in various hormones related to sexual function, such as GnRH, LH, testosterone and FSH. Studies examining the anti-aging effect on musculoskeletal function confirmed that these preparations had a positive effect, including improvement of gastrocnemius muscle and motor function.

## 4. Discussion

We reviewed in vivo (rodent) studies on the use of herbal medicines for anti-aging that were published in the last five years. A total of 41 articles were analyzed according to the described research selection criteria. As a result, it was confirmed that many single herbal medicines and complex herbal medicines have anti-aging effects on various organs and functions.

Four studies were conducted on the brain (cognition and emotion were separately classified), with two of those studies focusing on single herbal medicines and two focusing on complex herbal medicines. In addition to antioxidant effects, memory and motor functions were improved, and the effect on hippocampal neurogenesis was also confirmed. *Gastrodiae Rhizoma* is a single herbal medicine widely used for central nervous system (CNS) diseases, such as headache, dizziness, epilepsy and stroke [51]. *Lycium barbarum* and Modified Qiongyu paste have been used as promising anti-aging agents for a long time; in particular, a number of studies have been conducted on *Lycium barbarum* [52]. Therefore, these herbal medicines may be used for brain-related anti-aging.

Nine research papers have been published on learning and memory, cognition and emotion [12,13,14,15,16,17,18,19,20]. Seven of these focused on single herbal medicines [12,13,14,16,17,18,19], and two focused on complex herbal medicines [15,20]. *Scutellaria baicalensis* Georgi has traditionally been used to reduce fever and has been found to have anti-inflammatory, antiviral, antioxidant and antibacterial effects [53]. As this herb has been proven to be effective in memory impairment models in several studies [54,55,56], it can be widely used for anti-aging purposes through cognitive improvement and memory impairment prevention in the future. Dushen Tang is a prescription for the decoction of *Panax ginseng*. *Panax ginseng* is widely used as an effective herbal medicine, and many studies related to brain senescence have already been conducted [57]. Therefore, *Panax ginseng* alone can be used as an anti-aging remedy; it can also be added to a prescription in consideration of certain health conditions. The Morris water maze test is the most frequently used method to evaluate learning and memory, and some studies have confirmed the mechanism of reducing hippocampal neuronal damage and increasing synaptic density. Even changes at the mRNA level have been observed. These results provide the basis for the use of herbal medicines for cognitive improvement and dementia prevention.

The elderly population shows depressive symptoms that are different from those of general depression and may be accompanied by decreased concentration and memory, insomnia and personality changes. Kososan (Xiang-Su-San in Chinese; Hyangsosan in Korean), which was used in the study by Ito et al. [20], is a prescription that can be used for patients who experience no motivation, feeling down or poor digestive function. Depressive behavior was improved in the SAM8 mouse model, and this improvement was confirmed by observing the hippocampus and tau, which can be expected to help improve depression and cognitive function in the elderly.

There were a total of eight papers related to internal organs, such as the heart, liver and kidney, as well as the gastrointestinal tract [21,22,23,24,25,26,27,28]. There were seven studies on the use of a single herbal medicine [21,22,23,24,25,26,27] and one on complex herbal medicine [28]. Both studies were conducted in Taiwan [21,22], and it was confirmed that the administration of *Alpinia oxyphylla* Miq. reduced aging-related heart damage and improved heart function. *Alpinia oxyphylla* Miq. has been used for the purpose of enhancing cardiac function, and it has been shown to improve dementia through several experiments [58]. Recently, however, results of extending lifespan in a *Caenorhabditis elegans (C. elegans)* model have been reported [59]. Based on these results, it is thought that *Alpinia oxyphylla* Miq. may be useful for anti-aging in cardiac functions.

*Chrysanthemum indicum* Linne has been used for various diseases, such as high blood pressure and headache, and has been found to have hepatoprotective effects [60]. In a study by Zhang et al. [23], *Chrysanthemum indicum* Linne restored body weight and lowered ALT and AST levels in aging-induced mice. In addition, it alleviated the abnormal alterations in the structure and function of the brain and liver. Therefore, *Chrysanthemum indicum* Linne may be used to prevent aging in cases related to the liver. *Angelica sinensis* has been used for tonifying, invigorating blood, replenishing and treating female menstrual disorders [61]. *Angelica sinensis* is a hepatoprotective herbal medicine, and its mechanisms have been confirmed [62]. It is often used in combination with *Astragalus membranaceus*, and the combined effect of these two herbal medicines on nephrotic syndrome has been studied [63]. In a study by Mo et al. [26], *Angelica sinensis* improved various outcomes related to the liver and kidneys of aging-induced rats. Therefore, *Angelica sinensis* is effective for anti-aging, and it can be considered for the deterioration of liver and kidney function in the elderly.

There were two papers related to the gastrointestinal tract, one on single herbal medicine [27] and one on complex herbal medicine [28]. *Codonopsis pilosula* is a herbal medicine that strengthens the spleen and lungs [64]. Meng et al. [27] confirmed that *Codonopsis pilosula* improves gastrointestinal function and gastric histomorphological changes in aging mice. This is an experimentally proven clinical effect based on the medical literature, and it is possible to consider *Codonopsis pilosula* in elderly patients with reduced gastrointestinal function.

Regarding sexual function, there were three studies conducted on the use of single herbal medicines [31,33,34], and the remaining seven studies were conducted on the use of complex herbal medicines [30,32,35,36,37,38]. Testicular function, hypogonadism and ovarian function were shown to be restored by the administration of herbal medicine through sex hormones, morphological observation and gene level analysis using male and female aging models, respectively. The effect and mechanism of action of the Wuzi Yanzong recipe (Ojayeonjonghwan in Korean) on testicular dysfunction and hypogonadism were confirmed [35]. This prescription has been used to treat male infertility and has been reported to be effective against oligoasthenozoospermia [65]. Aging decreases sperm count and viability; moreover, changes in hormone secretion, such as testosterone and FSH, reduce sexual function. Samul-tang (Siwu-tang in Chinese; Shimotsu-to in Japanese) is a basic prescription that is mainly used for women with blood deficiency conditions and menstrual and uterine diseases. According to a study by Kim et al. [37], various mechanisms by which Samul-tang improved age-related decline in ovarian function have been identified. Yu Linzhu is a complex herbal medicine that is widely prescribed for infertility and habitual abortion, and its effects and mechanisms of restoring ovarian function have been confirmed [38]. It has also been confirmed that various herbal medicines inhibit sexual dysfunction. This is an important mechanism and effect for anti-aging.

There was a total of three articles that were classified by musculoskeletal function, one single herbal medicine study [39] and two complex prescription studies [40,41]. Juzentaihoto (Shi-quan-da-bu-tang in Chinese; Sipjeondaebo-tang in Korean) administration improved gastrocnemius muscle and motor function in SAMP8 mice [40]. Juzentaihoto is a complex herbal prescription that is mainly used for fatigue and loss of appetite. Recently, many studies on cancer have been conducted [66,67]. Therefore, if body function is weakened, it is possible to recover this function through the use of Juzentaihoto and to pursue its anti-aging effects.

In some articles, there were omissions, such as herbal medicine extraction methods [8,11,25,32,36,37,38,41,44,46], and in most studies, there was no description of the herbal medicine dosage calculation or aging-inducing drug dosage setting. In a model in which aging was induced by the administration of d-galactose, the administration concentration and duration were not certain; therefore, a guide may be necessary in future studies.

## 5. Conclusions

In this review, a variety of single and complex herbal medicines exhibited beneficial effects on anti-aging in various parts of the body and function. The common main mechanisms of action of herbal medicines are antioxidant and anti-inflammatory, and various mechanisms have been identified according to each organ and function. Learning and memory were shown to be improved through memory function tests and hippocampal neurogenesis, identifying the anti-aging mechanisms of the brain. Aging-associated cardiac damage, elevated liver enzyme levels and intestinal inflammation were decreased. The levels of GnRH, LH, testosterone, FSH, etc. improved. In addition, the mechanisms of action on each organ and its function were confirmed. Thus, the possibility that herbal medicine can be used in various ways for the purposes of anti-aging has been confirmed. In clinical practice, these herbal medicines can be used for treating the aging process.

However, since the experiments have been conducted using inconsistent models and methods, it is difficult to compare the results, which is a limitation of our analysis. Future studies to confirm and compare the anti-aging effects and mechanisms of various herbal medicines in the same model are recommended. Further investigation of the appropriate herbal medicine prescriptions and their components is also recommended. Accordingly, it is hoped that humans will live a long and healthy life by developing new natural products and conducting clinical research.

## Figures and Tables

**Table 1:** In vivo studies of herbal medicines for anti-aging.

Classification	Study (Author, year)	Country	Herbal Medicine (Part)	Herbal Extraction	Route of Administration	Dosage (Day)	Treatment Period	Animal Model (Age)	Induced Way	Sex	n/ Group	Outcomes and Mechanism
Brain												
	Xie et al., 2022 [8]	China	Modified Qiongyu paste	N/R	oral	0.3 g/kg, 0.6 g/kg, 1.2 g/kg	6 w	C57BL/6N mice (6–8 w)	D-gal (150 mg/kg/day for 6 w)	M	10	SOD (brain cortex) ↑ TNF-α (serum) ↓ IL-6 (serum) ↓
	Li et al., 2022 [9]	China	BaZiBuShen	water	oral	0.7 g/kg, 1.4 g/kg, 2.8 g/kg	65 d	ICR mice (8 w)	D-gal (120 mg/kg/day) and NaNO_2_ (90 mg/kg/day) for 3 m	M	14	memory and cognitive deficits ↓ motor function and grip strength ↑ GSH/GSSG, MDA, and TAC (brain) ↑ preserved mitochondrial function in cerebral cortex maintained telomerase activity and telomere length P53, caspase-3, Bax expressions ↓ Sirt6, p–HO–1, p-NRF2, PGC-1α, and Bcl-2 expressions↑
	Hsu et al., 2021 [10]	Taiwan	*Gastrodiae Rhizoma* (rhizome)	ethanol	intragastric	5 mg/kg, 20 mg/kg, 50 mg/kg	8 w	C57BL/6 mice (6 w)	D-gal (200 mg/kg/day for 8 w)	M	6	learning and memory abilities (nesting and burrowing test, and Morris water maze test) ↑ reversed the decreased CAT and SOD (brain) GSH-Px activity (the cortex and hippocampus) ↑ MDA ↓ hippocampal neurogenesis ↑ regulated the SH2B1-Akt pathway
	Ruíz-Salinas et al., 2020 [11]	Mexico	*Lycium barbarum* (fruits)	N/R	N/R	3 g/kg	60 d	Sprague-Dawley rats (18 m)	N/R	M	15	dendritic morphology in the PFC and hippocampus neurons ↑ Synaptophysin↑ Caspase 3 ↓ 3-nitrotyrosine ↓ Nrf2 ↓ ROS (PFC and hippocampus) ↓
Learning and memory												
	Sumiyoshi et al., 2021 [12]	Japan	*Anredera cordifolia* (leaves)	methanol	oral	N/R	31 w	SAMP8 mice (15 w)	N/R	M	9 or 10	faster acquisition and better retention in the Morris water maze task neuronal plasticity-related protein (hippocampal BDNF, NMDAR subunit, postsynaptic density protein-95, pCREB/CREB) ↑
	Wang et al., 2021 [13]	China	Dushen Tang	water	oral	0.3 g/kg	N/R	Sprague-Dawley rats (6 w)	D-gal (500 mg/kg/day for 7 w)	M	10	the spatial memory and learning abilities (Morris water maze test) ↑ neuronal damage in the hippocampus ↓ regulated the structure of the gut microbiota
	Xia et al., 2019 [14]	China	*Eclipta prostrata* (leaves)	water	oral	50 mg/kg, 100 mg/kg, 200 mg/kg	3 w	Sprague-Dawley rats (30 w)	D-gal (100 mg/kg/day for 6 w)	M	10	the spatial memory and learning abilities (Morris water maze test) ↑ neuronal damage in the hippocampus↓ SOD, CAT, GSH-Px, GR ↑ iNOS, NO ↓ dopamine, norepinephrine, serotonin (5-HT) ↑
	Zhao et al., 2020 [15]	China	Guilingji	suspension	oral	37.5 mg/kg, 75 mg/kg, 150 mg/kg	4 w	Sprague-Dawley rats (23 m)	N/R	M	10	the spatial memory and learning abilities (Morris water maze test) ↑ SOD, CAT and GSH-Px ↑ MDA ↓ Ach ↑ AchE ↓ ^1^H-NMR-based serum metabolomics
	Zhao et al., 2018 [16]	China	*Scutellaria baicalensis* Georgi (root)	ethanol	oral	100 mg/kg/, 200 mg/kg	10 w	Sprague-Dawley rats	D-gal (100 mg/kg/day for 10 w)	M	10	the spatial and learning memory (open-field test and Morris water maze test) ↑ SOD, CAT, GPx ↑ MDA ↓ histological abnormalities of hippocampus neurons ↓
Cognition												
	Gray et al., 2018 [17]	USA	*Centella asiatica* (leaves)	water	oral	2 g/L	2 w	CB6F1 mice (20 m)	N/R	M, F	18 (M + F)	performance in the Object Location Memory task ↑ performance in the Novel Object Recognition Task ↑ synaptic density in the hippocampus ↑
	Fainanta et al., 2022 [18]	Thailand	*Pueraria mirifica* (N/R)	ethanol	oral	100 mg/kg	2 m	Sprague-Dawley rats (androgen-deficient, 2 m)	orchidectomized	M	13	impairment of spatial learning behavior and memory capacity was prevented maintain synaptic function and structure and suppress neurofibrillary tangles mitigated the increased *Tau3* and *Tau4* mRNA levels
	Zhao et al., 2020 [19]	China	*Zanthoxylum bungeanum* Maxim (N/R)	water or volatile oil	oral	450 mg/kg	48 d	Kunming mice	D-gal (500 mg/kg)	M	10	impaired memory was alleviated prevented hippocampal neuron damage MDA (brain) ↓ upregulated Nrf2 and HO-1 the expression ratio of Bcl2/Bax ↑
Emotion												
	Ito et al., 2022 [20]	Japan	Kososan, Hachimijiogan	water	oral	1.0 g/kg	13 w	SAMP8 mice (7 w)	N/R	M	N/R	depression-like behaviors (the tail suspension test, sucrose preference test, and open field test) ↓ hippocampal neuroinflammation ↓ tau accumulation ↓ IL-6 and MCP-1 ↓
Internal organs												
Heart	Chang et al., 2021 [21]	Taiwan	*Alpinia oxyphylla* Miq. (fruits)	water	oral	100 mg/kg	4 w	Wistar-Kyoto rats (20 w)	D-gal (150 mg kg/day for 8 w)	M	6	aging associated cardiac damages ↓ cardiac performance ↑
Heart	Chang et al., 2019 [22]	Taiwan	*Alpinia oxyphylla* Miq. (fruits)	water	oral	50 mg/kg, 100 mg/kg, 150 mg/kg	8 w	Sprague-Dawley rats (8 w)	D-gal (150 mg kg/day for 8 w)	M	N/R	collagen deposition and cardiac fibrosis ↓
Liver and brain	Zhang et al., 2019 [23]	China	*Chrysanthemum indicum* Linne (flowers and buds)	carbon dioxide fluid	oral	100 mg/kg, 150 mg/kg, 300 mg/kg	8 w	Kunming mice (N/R)	D-gal (200 mg/kg)	M	10	body weight ↑ attenuated the decline of thymus and spleen indexes elevated levels of ALT and AST ↓ alleviated abnormal alterations in structure and function of brain and liver ↓ renewing normal antioxidant enzymes activities (SOD, CAT, GSH-Px) MDA accumulation ↓ IL-1β, IL-6, TNF-α ↓ attenuated the increase of Bax/Bcl-2 ratio and cleaved caspase-3 activation in the liver and brain
Liver	Kim et al., 2023 [24]	Korea	*Acanthopanax senticosus* (stem and root)	ethanol	oral	7 mg	24 w	Sprague-Dawley rats (24 w)	N/R	M	10	leukocyte telomere length ↑ AST, ALT ↓
Liver	Tewari et al., 2020 [25]	Poland	*Trigonella foenum-graecum* L. (seeds)	N/R	oral	0.125 g, 0.250 g	4 w	Swiss mice (12 m)	N/R	M	6	SOD ↑ glutathione reductase ↓ GSH-Px ↓ total polyphenols↑ free radical scavenging properties↑
Liver and kidney	Mo et al., 2018 [26]	China	*Angelica sinensis* (root)	carbon dioxide fluid	oral	20 mg/kg, 40 mg/kg, 80 mg/kg	8 w	Kunming strain mice (8 w)	D-gal (200 mg/kg/day for 8 w)	M	N/R	the organ index ↑ functional parameters ↑ the hepatic and renal MD ↓ gene expressions of hepatic and renal Cu, Zn-SOD, CAT, and GPx ↑ iNOS, COX-2, IκBα, p-IκBα, and p65 ↓ IκBα ↑ ameliorated the histological deterioration
Gastrointestinal tract												
	Meng et al., 2021 [27]	China	*Codonopsis pilosula* (root)	water	intragastric	5 g/kg, 10 g/kg, 15 g/kg	6 w	Kunming mice (2 m)	D-gal (50 g/kg/day)	M, F	20	weight and thymus index ↑ D-xylose absorption ↑ motilin secretion ↑ reversed the changes of gastric histomorphology
	Piao et al., 2020 [28]	China	Fufang Zhenzhu Tiao Zhi	alcohol and water	oral	1.0 g/kg	12 w	C57BL/6J mice (22 m)	naturally aging	F	6	intestinal inflammation ↓ telomerase activity ↑ partially reversed the fecal metabolites abnormalities restored the disorders of intestinal flora
Sexual function												
	Ding et al., 2022 [29]	China	Guilingji	suspension	intragastric	37.5 mg/kg, 75 mg/kg, 150 mg/kg	8 w	Sprague-Dawley rats (6 w)	D-gal (300 mg/kg/day for 8 w)	M, F	6	the mount and ejaculation latency levels ↑ testicular morphology improved GnRH and LH levels improved
testicular function	Zhao et al., 2019 [30]	China	Guilingji	suspension	intragastric	37.5 mg/kg, 75 mg/kg, 150 mg/kg	4 w	Sprague-Dawley rats (23 m)	naturally aging	M	10	Weights of the testicles ↑ T concentration ↑ Morphologic abnormality of testicular tissues: improved Urinary levels of alanine, pantothenate, phenylalanine, β-hydroxybutyrate and pyruvate ↓
	Ku et al., 2020 [31]	Korea	Korean Red Ginseng (root)	water	oral	50 mg/kg	4 w	C57BL/b inbred mice (12 m)	N/R	M	10	sperm production and sperm motility ↑ testosterone and FSH (serum) ↑ VEGF ↑ spermatogenesis-related genes (inhibin-α, nectin-2, and CREB) ↑
	Zhao et al., 2019 [32]	China	Wuzi Yanzong recipe	N/R	oral	1 g/kg, 4 g/kg	4 m	Sprague-Dawley rats (16 m)	N/R	M	10	the testicular weight and index ↑ sperm count and viability ↑ testosterone ↑ estradiol ↓ activated the onset of ERS germ cell apoptosis ↓
hypogonadism	Jung et al., 2018 [33]	Korea	*Dendropanax morbiferus* H.Lév (leaves)	water	oral	50 mg/kg, 100 mg/kg, 200 mg/kg	4 w	Sprague-Dawley rats (6–7 m)	N/R	M	5	improved physical tests (rotarod, treadmill, and swimming tests) testosterone, LH ↑ TG and LDL cholesterol ↓ testicular spermatogenesis ↑
	Jeong et al., 2020 [34]	Korea	*Lycium chinense* P. Mill (fruits)	ethanol	oral	150 mg/kg, 300mg/kg	6 w	Sprague-Dawley rats (18 m)	N/R	M	6	sperm counts and motility testosterone ↑ androgen receptor expression (testis and prostate) ↑ SOD ↑ 8-OHdG ↓ Bcl-2 ↑ apoptotic activator (BAX) ↓ phosphorylated Akt and ERK ↑
	Choi et al., 2019 [35]	Korea	Modified Ojayeonjonghwan	ethanol	oral	200 mg/kg, 400 mg/kg	4 w	Sprague-Dawley rats (18 m)	N/R	M	6	the weights of testis and epididymis ↑ testosterone (serum) ↑ SOD ↑ 8-OHdG ↓ upregulated androgen receptor expression in testicular tissue
ovarian function	Zhang et al., 2018 [36]	China	Heyan Kuntai Capsule	N/R	intragastric	0.3 g/kg, 0.9 g/kg, 2.7 g/kg	4 w	C57BL/6J mice (11 m)	naturally aging	F	5	the total number of follicles ↑ the number of primordial and primary follicles ↑ suppressed the apoptosis of follicles did not alter serum estrogen concentrations
	Kim et al., 2021 [37]	Korea	Samul-tang	N/R	oral	2.5 g/kg 5 times per week	4 w	BALB/c mice (40 w)	N/R	F	7	AMH and FSH (serum) ↑ prevented age-related ovarian follicle loss Quality of oocytes and blastocysts were enhanced reversed aged-induced changes in mRNA expression (ovary) triggered changes in aging-related genes
	Yang et al., 2021 [38]	China	Yu Linzhu	N/R	intragastric	0.3 mL	6 w	BALB/c mice (9 m)	naturally aging	F	10	the ovarian area recovered the ovarian blood flow improved the ovarian volume ↑ the degree of adhesion ↓ the infiltration of ovarian interstitial lymphocytes ↓ the zona pellucida recovered FSH and LH ↓ E2 and AMH ↑ ROS, MDA ↓ GSH-Px ↑ mitochondrial function of oocytes improved
Musculoskeletal function												
Bone	Li et al., 2019 [39]	China	*Fructus Ligustri Lucidi* (fruits)	water	intragastric	4.9 g/kg/day	65 d	ICR mice	D-gal (120 mg/kg/day) and NaNO_2_ (90 mg/kg/day) for 3 m	M	10	revealed a non-osteoporotic bone phenotype the memory and cognitive function ↑ MDA, 8-OH-dG, Nox4 ↓ TAC, GSH/GSSG ↑ the regulation of gut microbiota diversity
muscle and motor	Morita et al., 2021 [40]	Japan	Juzentaihoto	water	oral	4%	18 w	SAMP8 mice (18 w)	N/R	M	6, 7	gastrocnemius muscle and extensor digitorum longus weights ↑ gastrocnemius muscle fiber cross-sectional areas ↑ motor function (Rota-rod test) ↑ IGF-1(serum), mRNA Sirt1 ↑ TNF-α, IL-6 ↓ mRNA levels of Atrogin1 and MuRF1 (gastrocnemius) ↓
muscle and motor	Amitani et al., 2022 [41]	Japan	Ninjin’yoeito	N/R	oral	3%, 5%	30 d	klotho-hypomorphic (kl/kl) mice (4 w)	N/R	M	11	survival rate ↑ free walking, rotarod, and spontaneous activity test ↑ triceps surae muscles weight ↑ bone strength ↑ telomere content ↑ age-related histological declines in heart, lung, thymus, testis, bone tissue, muscles and age-related motor dysfunction were improved
Others												
Skin	Orita et al., 2020 [42]	Japan	Hochu-ekki-to	water	oral	1.0 g/kg, 3 times per week	2 y	hairless mice (8 w)	naturally aging	N/R	10	moisture retention, skin hydration, and the generation of wrinkles: improved vitamin A, vitamin C, collagen type I, collagen type III, fibroblasts, and hyaluronic acid levels in the skin ↑ ROS ↓
Skin and sexual organs	Hiramoto et al., 2020 [43]	Japan	*Momordica charantia* (fruits)	water	oral	50 mg/kg, 3 times per week	2 y	bred hairless mice (8 w)	naturally aging	M, F	10	improved moisture retention, hydration, thickness, and reduced wrinkle score cell apoptosis (ovaries and testes) ↓ MMP-1 and hyaluronidase 2 (skin) ↓ IL-33 ↑
Lifespan extension	Wang et al., 2019 [44]	Japan	Rikkunshito	N/R	oral	N/R	① 12 w ② 14 w ③ 15 w	① wild-type and NPY knockout mice (82–89 w) ② inbred C57BL/6 mice (14–16 w) ③ C57BL/6 mice (16–18 w)	① N/R ② 3-nitropropionic acid injection to induce oxidative stress ③ doxorubicin injection to induce oxidative stress	M, F	① 19, 20 ② 18, 19 ③ 13, 14	① no significant effect on lifespan body weight, white adipose tissue weight and brown adipose tissue weight ↓ ghrelin levels ↑ ② upregulated anti-oxidative gene expression in the liver ③ plasma ghrelin concentration ↑
Physiology (geriatric syndromes)	Matsubara et al, 2022 [45]	Japan	Ninjin’yoeito	water	oral	1%, 3%	11 w	C57BL/6 mice (88 w)	N/R	M	10–15	rectal temperature ↑ forelimb grip strength ↑ self-care motivation (sucrose splash test) ↑
Gut microbiota and the metabolome	Luo et al., 2020 [46]	China	FuFang Zhenshu TiaoZhi	N/R	oral	1.0 g kg	12 w	C57BL/6JNarl mice (20 m)	N/R	N/R	6	the autonomous activity and the motor coordination ability ↑ glucose, lipids ↓ TNF-α, IL-6 ↓ the diversity and abundance of gut microbiota ↑ regulate the structure of gut microbiota
Metabolomic Analysis	Zhao et al., 2018 [47]	China	*Glycyrrhiza glabra* (Licorice) (root)	water	oral	1 g/kg, 10 g/kg	7 w	Sprague-Dawley rats (7 w)	D-gal (300 mg/kg/day for 7 w)	M	10	taurine metabolic pathway was significantly correlated with the ageing process taurine, CDO1 and CSAD ↑
Metabolomic Analysis	Xi et al., 2021 [48]	China	Liuwei Dihuang decoction	water	oral	9.75 g/kg	30 d	ICR mice (20 m)	naturally aging	M	10	the organ index ↑ weight-bearing swimming time ↑ regulated the expression level of 11 aging-associated metabolites

N/R, not reported; mg/kg, milligram/kg; g, gram; d, day; w, week; m, month; y, year; M, male; F, female; ↑, increase; ↓, decrease; Ach: acetylcholine; AChE: acetylcholinesterase; ALT: alanine aminotransferase; AMH: anti-Müllerian hormone; AST: aspartate aminotransferase; Bax: Bcl-2 associated x protein; Bcl-2: B-cell lymphoma-2; BDNF: brain-derived neurotropic factor; CAT: catalase; CDO1: cysteine dioxygenase type I; CREB: cAMP-response element binding; CSAD: cysteine sulfinic acid decarboxylase; D-gal: D-galactose; ERS: endoplasmic reticulum stress; E2: estrogen; FSH: follicle stimulating hormone; GnRH: gonadotropin-releasing hormone; GR: glutathione reductase; GSH: reduced glutathione; GSH-Px: glutathione peroxidase; GSSG: oxidized glutathione disulfide; HO-1: heme oxygenase 1; ICR: Institute of Cancer Research; IL: interleukin; LH: luteinizing hormone; MDA: malondialdehyde; MMP: matrix metalloprotease; NaNO_2_: sodium nitrite; NMDAR: NMDA-receptor; NPY: neuropeptide Y; Nrf2: nuclear factor erythroid 2-related factor 2; PFC: prefrontal cortex; PGC-1α: peroxisome proliferator activated receptorγcoactivator-1α; p-NRF2: phospho-nuclear factor erythroid 2-related factor 2; SAMP8: senescence-accelerated mouse-prone 8; Sirt6: Sirtuin 6; SOD: superoxide dismutase; TAC: total antioxidant capacity; TG: triglyceride; TNF-α: tumor necrosis factor-α; VEGF: vascular endothelial growth factor; 8-OHdG: 8-hydroxy-20-deoxyguanosine.

**Table 2:** Composition of multi-compound herbal prescriptions.

Study (Author, Year)	Herbal Prescriptions	Components
Xie et al., 2022 [8]	Modified Qiongyu paste	*Panax ginseng*, *Poria cocos*, *Rehmannia glutinosa*, *Cistanche deserticola*, *Salvia miltiorrhiza*.
Li et al., 2022 [9]	BaZiBuShen	*Cuscuta chinensis* Lam., *Lycium barbarum* L., *Epimedium brevicornu* Maxim., *Schisandra sphenanthera* Rehder & E.H. Wilson, *Cnidium monnieri* (L.) Cuss, *Rosa laevigata* Michx., *Rubus idaeus* L., *Allium tuberosum* Rottler ex Spreng., *Morinda officinalis* F.C. How, *Cistanche deserticola* Y.C. Ma, *Rehmannia glutinosa* (Gaertn.) DC., *Cyathula officinalis* K.C. Kuan, *Panax ginseng* C. A. Mey., Young unossified hairy antler of male Cervus, Nippon Temminck or. *Cervus elaphus* Linnaeus (Cervidae)., Marine teleost fish, *Melia azedarach* L.
Wang et al., 2021 [13]	Dushen Tang	*Panax ginseng* C.A. Mey
Zhao et al., 2020 [15] Ding et al., 2022 [29] Zhao et al., 2019 [30]	Guilingji	*Ginseng Radix et Rhizoma Rubra*, *Cervi Cornu Pantotrichum*, *Hippocampus*, *Lycii Fructus*, *Caryophylli Flos*, *Manis Squama*, *Passeris Meddula Achyranthis Bidentatae Radix*, *Cynomorii Herba*, *Rehmanniae Radix Praeparata*, *Psoraleae Frutus*, *Cuscutae Semen*, *Eucommiae Cortex*, *Spirferis Fossilia*, *Cistanches Herba*, *Glycyrrhizae Radix et Rhizoma*, *Asparagi Radix*, *Epimedii Folium*, *Halitum and Amomi Fructus*
Ito et al., 2022 [20]	Kososan	*Cyperus rhizome*, *Perilla herb*, *Citrus unshiu peel*, *Glycyrrhiza*, *Ginger*
Ito et al., 2022 [20]	Hachimijiogan	*Rehmannia root*, *Alisma rhizome*, *Poria sclerotium*, *Dioscorea rhizome*, *Cornus fruit*, *Moutan bark*, *Cinnamon bark*, *Aconite root*
Piao et al., 2020 [28] Luo et al., 2020 [46]	Fufang Zhenzhu Tiao Zhi	*Citri sarcodactylis fructus*, *Ligustri lucidi fructus*, *Salviae miltiorrhizae radix et rhizoma*, *Notoginseng radix et rhizoma*, *Coptidis rhizoma*, *Atractylodis macrocephalae rhizoma*, *Cirsii japonici herba et radix*, *Eucommiae cortex*
Zhao et al., 2019 [32]	Wuzi Yanzong recipe	*Plantaginis semen, Rubi fructus*, *Schisandrae chinensis fructus*, *Lycii fructus, Cuscutae semen*
Choi et al., 2019 [35]	Modified Ojayeonjonghwan	*Cornus officinalis Sieb. et Zucc.*, *Lycium chinense Miller*, *Rubus coreanus Miquel, Cuscuta chinensis Lam*, *Schisandra chinensis Baillon*
Zhang et al., 2018 [36]	Heyan Kuntai Capsule	*Radix Rehmanniae praeparata*, *Radix Paeoniae Alba*, *Colla Corii Asini*, *Rhizoma Coptidis*, *Radix Scutellariae*, *Poria*
Kim et al., 2021 [37]	Samul-tang	*Paeonia lactiflora*, *Liqusticum striatum*, *Rehmannia glutinosa*, *Angelica gigas*.
Yang et al., 2021 [38]	Yu Linzhu	*Rehmanniae Radix Preparata*, *Cuscutae Semen*, *Gingseng Radix*, *Atractylodis Rhizoma alba*, *Poria cocos, Paeoniae Radix*, *Eucommiae Cortex, Cervi Cornus Colla.*, *Angelicae Gigantis Radix*, *Cnidium officinale*, *Glycyrrhizae Radix*
Morita et al., 2021 [40]	Juzentaihoto	*Astragali Radix*, *Atractylodis Lanceae Rhizoma*, *Cinnamomi Cortex*, *Angelica Radix*, *Rehmanniae Radix*, *Ginseng Radix*, *Paeoniae Radix*, *Poria, Cnidii Rhizoma*, *Glycyrrhizae Radix*
Amitani et al., 2022 [41] Matsubara et al., 2022 [45]	Ninjin’yoeito	*Rehmannia root*, *Japanese angelica root*, *Atractylodes rhizome*, *Poria sclerotium, Ginseng*, *Cinnamon bark*, *Polygala root*, *Peony root*, *Citrus unshiu peel*, *Astragalus root*, *Glycyrrhiza*, *Schisandra fruit*
Orita et al., 2020 [42]	Hochu-ekki-to	*Astragali Radix*, *Astractylodis Rhizoma*, *Ginseng Radix*, *Angelicae Radix*, *Bupleuri Radix*, *Zizyphi Fructus*, *Auranti Nobilis Pericarpium*, *Glycyrrhizae Radix*, *Cimcifugae Rhizoma*, *Zingiberis Rhizoma*
Wang et al., 2019 [44]	Rikkunshito	*Atractylodes lancea rhizome*, *ginseng*, *pinellia tuber*, *Poria sclerotium*, *jujube*, *citrus unshiu peel*, *Glycyrrhiza and ginger*
Xi et al., 2021 [48]	Liuwei Dihuang decoction	*Rehmannia glutinosa*, *Cornus officinalis*, *Chinese yam*, *Poria cocos*, *Moutan bark*, *Alisma*

## Data Availability

No new data were created or analyzed in this study. Data sharing is not applicable to this article.
